# LoRaWAN Meets ML: A Survey on Enhancing Performance with Machine Learning

**DOI:** 10.3390/s23156851

**Published:** 2023-08-01

**Authors:** Arshad Farhad, Jae-Young Pyun

**Affiliations:** Wireless and Mobile Communication System Laboratory, Department of Information and Communication Engineering, Chosun University, Gwangju 61452, Republic of Korea; arshad@chosun.ac.kr

**Keywords:** LoRa, LoRaWAN, Internet of Things (IoT), machine learning (ML), resource management, spreading factor (SF), transmission power (TP), simulation, artificial intelligence, deep learning, reinforcement learning, dataset

## Abstract

The Internet of Things is rapidly growing with the demand for low-power, long-range wireless communication technologies. Long Range Wide Area Network (LoRaWAN) is one such technology that has gained significant attention in recent years due to its ability to provide long-range communication with low power consumption. One of the main issues in LoRaWAN is the efficient utilization of radio resources (e.g., spreading factor and transmission power) by the end devices. To solve the resource allocation issue, machine learning (ML) methods have been used to improve the LoRaWAN network performance. The primary aim of this survey paper is to study and examine the issue of resource management in LoRaWAN that has been resolved through state-of-the-art ML methods. Further, this survey presents the publicly available LoRaWAN frameworks that could be utilized for dataset collection, discusses the required features for efficient resource management with suggested ML methods, and highlights the existing publicly available datasets. The survey also explores and evaluates the Network Simulator-3-based ML frameworks that can be leveraged for efficient resource management. Finally, future recommendations regarding the applicability of the ML applications for resource management in LoRaWAN are illustrated, providing a comprehensive guide for researchers and practitioners interested in applying ML to improve the performance of the LoRaWAN network.

## 1. Introduction

The Internet of Things (IoT) is a rapidly growing field that involves connecting a wide range of devices to the Internet to enable communication and data exchange between them. IoT enables seamless integration of the physical and digital worlds, revolutionizing various domains such as healthcare, transportation, agriculture, and industrial automation [1,2,3,4]. In IoT connectivity, several technologies have emerged to address the diverse requirements of IoT applications. These technologies comprise Long-Range Wide Area Networks (LoRaWAN), SigFox, Narrowband (NB)-IoT, Weightless, and Long Term Evolution for Machines (LTE-M) [5,6,7,8]. The key features of these IoT technologies are illustrated in Table 1. Sigfox offers a simple and low-cost deployment, while NB-IoT leverages the existing cellular infrastructure and provides higher data rates. The Weightless protocol provides flexibility and scalability, and LTE-M supports enhanced mobility and coverage. LoRaWAN [9] is among the leading low-power wide area network (LPWAN) technologies that have gained significant attention recently due to its ability to provide long-range communication with low power consumption. As a result, it has been extensively adopted by academia and industries for the IoT.

Long Range (LoRa) is the physical layer (PHY) primarily based on chirp spread spectrum (CSS) modulation, making it capable of achieving long-range and low power consumption [39]. LoRaWAN is the medium access control layer (MAC) responsible for efficiently managing communication between LoRa end devices (ED) and gateways (GW). In addition, LoRaWAN offers features such as adaptive data rate (ADR) for efficient resource management, bi-directional communication, and strong security, making it a robust and scalable solution for IoT deployments [40]. With their long range, ultra-low power consumption, and efficient network management, LoRa and LoRaWAN are revolutionizing the IoT landscape, empowering businesses and industries with seamless connectivity and enabling innovative IoT applications across various sectors [41].

### 1.1. Existing Surveys on LoRa/LoRaWAN and Motivation

The specification of LoRa provides a detailed overview of the PHY and LoRaWAN features along with the ADR, retransmission procedures, and other features [42]. However, the decision-making of resource parameters configuration and optimal allocation to EDs [e.g., SF, bandwidth (BW), coding rate (CR), and transmission power (TP)] is left open to developers and academic researchers, allowing them to create and develop solutions for IoT applications.

In recent years, the LoRa/LoRaWAN has been surveyed in various aspects, such as ADR optimizations, mobility management, simulation tools, routing, security, etc., highlighting advantages, disadvantages, and future recommendations, as illustrated in Table 2. Table 2 presents the existing surveys and tutorials with the main focus and brief description of the topics covered on LoRa/LoRaWAN. In addition, the current surveys and tutorials in Table 2 are not focusing on the resource allocation issue addressed using machine learning (ML) methods for the LoRa/LoRaWAN. Therefore, this survey fills the stated gap by presenting a constructive and comprehensive review of the use of ML in LoRa/LoRaWAN.

### 1.2. Methodology

**Figure 1:**
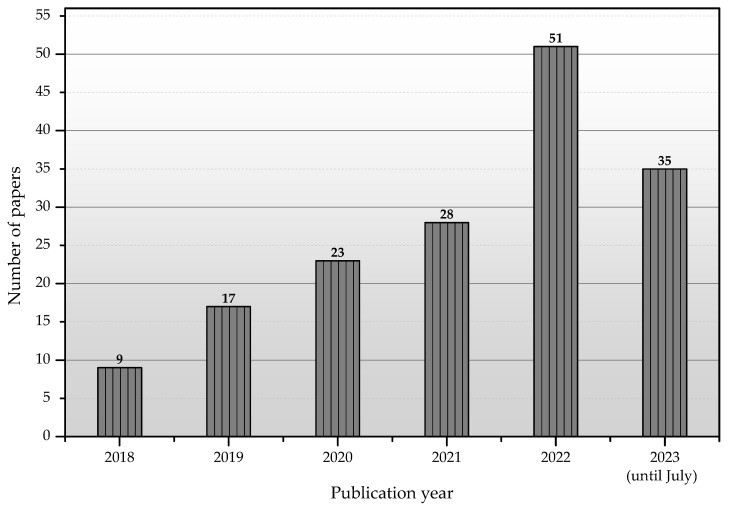
Number of published articles found in the existing literature for improving the performance of LoRaWAN using ML applications.

We started with a systematic literature review methodology for this survey on improving LoRaWAN performance with ML, as illustrated in [85]. We first comprehensively searched the abstracts of all papers on IEEE Xplore, ACM, Elsevier, Wiley, and MDPI databases for LoRa/LoRaWAN and AI/ML/DL/RL. The search yielded approximately 148 papers, which include all aspects of LoRa/LoRaWAN resolved through ML techniques, as illustrated in Figure 1.

However, this survey only focuses on the performance improvement of LoRaWAN achieved using efficient resource management (e.g., channel allocation, SF, TP, and BW) through ML. Out of 148 papers, we found 36 papers dealing with resource management issues. This number also includes manually adding a small number of papers not found by our initial search, using backward reference searching and cross-citation techniques.

### 1.3. Scope and Contribution of the Survey

In contrast to published surveys and tutorials highlighted with a brief description of the main topics covered in Table 2, which present many characteristics or provide a comprehensive evaluation of the LoRa and LoRaWAN communication systems, where a comparison with other LWPAN technologies, potentials of both LoRa and LoRaWAN, ADR optimization/enhancement techniques, interference/collision mitigation, and available simulators for LoRa/LoRaWAN are the major topics, our survey mainly focuses on resource allocation issue addressed through ML for improving LoRaWAN performance.

The contribution of this survey, compared to the surveys and tutorials presented in Table 2, is as follows:

1. We provide a systematic overview of the different areas of LoRaWAN performance where ML/DL/RL has been applied. We discuss the core LoRaWAN issues that can be addressed with ML/DL/RL and provide examples of how ML/DL/RL has been used to address these issues;
2. We discuss the publicly available LoRaWAN frameworks, which can potentially be applied for dataset collection. A comprehensive study has been carried out to highlight the best features for efficient resource allocation and the ML/DL/RL methods for improving LoRaWAN performance;
3. We extensively provide a discussion on the Network Simulator-3-based ML/DL/RL frameworks that could be utilized for efficient resource allocation with comprehensive scenarios;
4. We identify open challenges in each area of LoRaWAN performance, discuss future research directions concerning resource allocation, and highlight potential benefits of ML for improving LoRaWAN performance.

### 1.4. Structure of the Survey

Section 2 highlights the core features of LoRa and LoRaWAN. Section 3 presents state-of-the-art methods for improving LoRaWAN performance using ML, DL, and RL. Section 4 elaborates on the existing LoRaWAN frameworks that could be utilized for dataset collection, discusses the required features, and highlights the best suitable ML methods for resource allocation concerning the features. Section 5 discusses the publicly available datasets utilized for various LoRaWAN deployments and IoT applications. Section 6 presents the existing publicly available ns-3-based ML frameworks and discusses how to utilize them for improving the performance of LoRaWAN. Section 7 presents a detailed discussion and highlights the potentials and limitations of the resource management ML methods applied on EDs and NS sides. Section 8 elaborates on the open research opportunities regarding efficient resource management, whereas Section 9 provides concluding remarks on this survey paper.

## 2. Core Features of LoRa and LoRaWAN

This section briefly presents the core features of LoRa and LoRaWAN.

### 2.1. LoRa-Long Range

LoRa [86] is a radio frequency (RF) modulation technology that defines the PHY layer features for long-range communications. LoRa is a proprietary PHY layer modulation based on CSS modulation to achieve long-range communication [87]. CSS is a subset of Direct-Sequence Spread Spectrum (DSSS), helping the GW to recover a weak signal and achieve high sensitivity, enabling increased coverage at a lower data rate (DR) [88,89]. In addition, LoRa utilizes five configurable resource parameters, i.e., SF, TP, CR, BW, and carrier frequency (CF), to fine-tune the link performance and energy consumption [56,74,79,90,91]. These configurable resource parameters of LoRa communication are discussed here.

#### 2.1.1. Spreading Factor (SF)

The number of bits encoded in a symbol by LoRa is an adjustable resource parameter known as the SF. LoRa operates in six SFs (i.e., $SF_{7}$∼$SF_{12}$), utilized by the ED during uplink (UL) transmission. To transmit a UL packet, the EDs select a random channel using ALOHA channel access mechanism [92]. Furthermore, the choice of SF an ED utilizes during the communication plays a significant role for different reasons: a higher SF (e.g., 11, 12) complies with a high distance coverage; however, it indicates a low DR and high time-on-air (ToA) [67,93,94]. For example, the ToA for a packet size of 51B and 1% duty cycle (DC), considering the EU region (i.e., 868 MHz), is illustrated in Table 3. Table 3 is computed using The Things Network (TTN) community network platform, where the TTN has utilized the LoRaWAN regional parameters [9], consisting of the duty-cycled limited transmissions to comply with the European Telecommunications Standards Institute (ETSI) regulations. In the EU region, the ETSI imposes DC limitations, where LoRaWAN complies with a maximum DC of 1%. The TTN uses a fair access policy (FAP) [95], allowing ED to send data to GW for at most 30 s of ToA and ten downlink messages (including acknowledgments for confirmed packets) per ED per 24 h [96,97]. Based on the TTN network, $SF_{11}$ and $SF_{12}$ are only allowed when ADR is enabled. Increasing the SF by one step doubles the ToA (for the same BW). It also indicates that a single transmission on $SF_{10}$ takes more time than 6 on $SF_{7}$, or may need about the same ToA as 3 on $SF_{7}$, $SF_{8}$, and $SF_{9}$ combined. As a consequence of this behavior, the use of ADR or blind ADR is suggested for SF and TP adjustment [98,99,100].

#### 2.1.2. Transmission Power (TP)

In LoRa, TP is an adjustable parameter with a step of 2, ranging from 2 to 14 dBm. TP is controlled by the ADR, implemented at the ED and NS sides to control the energy consumption of EDs [103,104].

#### 2.1.3. Coding Rate (CR)

LoRa uses forward error correction (FEC) to improve the reliability of wireless transmissions. FEC adds redundant bits to the data, which can be used to correct errors occurring during transmission. The CR determines the redundancy added to the data. The smaller the CR, the more redundant bits are added, and the more reliable the transmission will be. However, a smaller CR will also increase the time it takes to transmit the data. The CR can be chosen among 4/5, 4/6, 4/7, and 4/8. The smallest CR, 4/8, provides the best reliability but takes the longest ToA to transmit the data. The largest CR, 4/5, provides the least reliability but sends the data the fastest. The choice of CR depends on the application that requires high reliability, such as industrial automation. A large CR should be used for applications that require fast data transmission, such as asset tracking.

#### 2.1.4. Carrier Frequency (CF)

CF in LoRaWAN is the frequency at which a LoRa ED transmits data toward GW. It is typically selected from a range of frequencies in a particular region. The CF affects the capacity and power consumption of ED. For example, the LoRa CF can be programmed in steps of 61 Hz between 137 MHz to 1020 MHz. However, depending on the particular LoRa chip, this range may be limited to 860 MHz to 1020 MHz [103]. LoRa supports different ISM frequencies (in MHz), namely EU863-870 (Europe), US902-928 (North America), EU433 (Asia), CN470-510, CN779-787 (China), AU915-928 (Australia), KR920-923 (Korea), and IN865-867 (India) [9].

#### 2.1.5. Bandwidth (BW)

LoRa operates in three BW: 125, 250, and 500 kHz. The BW is determined by the regional parameters, as specified in the LoRaWAN specifications [9]. A LoRa-modulated signal comprises $2^{SF}$ chips spread over the available BW. The SF parameter controls the spreading BW and the signal sensitivity to noise. A larger SF value results in a wider spreading of BW and lower sensitivity to noise. However, it reduces the DR.

### 2.2. Long Range Wide Area Network (LoRaWAN)

**Figure 2:**
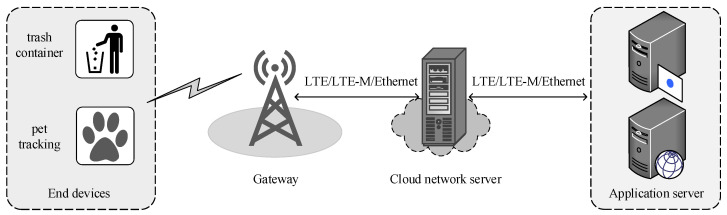
LoRaWAN network.

LoRaWAN defines MAC layer features as consisting of a star-of-stars topology comprising many EDs, GW, NS, and application servers, as shown in Figure 2. The EDs in LoRaWAN network are classified as Class *A*, Class *B*, and Class *C* [105]. Class *A* EDs are battery-powered and consume ultra-low energy. These EDs are bi-directional and receive acknowledgment (ACK) from NS with two available receive windows (RXs). Class *B* EDs are also battery-powered and provide bi-directional communication. These EDs support unicast and multicast transmission, though they have more RXs and are synchronized with a beacon frame transmitted by the GW after a certain time. Finally, Class *C* EDs use more power and listen all the time, excluding the transmission time. Among these EDs classes, Class *A* EDs deal with sensors and are implemented in IoT applications, owing to their energy efficiency and bi-directional communications [79]. Furthermore, LoRaWAN supports two communication modes: confirmed and unconfirmed.

#### 2.2.1. Confirmed Mode

In LoRaWAN, the ED initiates data transmission with an SF and TP. The SF and TP are allocated by the NS using the ADR mechanism, as defined in [99,106,107,108,109,110,111,112,113]. The ADR determines the values of SF and TP based on the highest SNR value of the last 20 packets received at the NS. The NS reduces the SF and increases or decreases the value of TP by 2 to reduce energy consumption. However, the newly adapted SF and TP might not successfully deliver the packet to the NS. Therefore, in confirmed mode, the ED utilizes a recovery ADR based on the retransmission procedure on the ED side. When the retransmission counter is a multiple of two, the ED increases its SF, and a TP of 14 dBm is adopted at the time of packet transmission [114]. It increases the chances of successfully delivering a packet to the NS with increased energy consumption costs.

#### 2.2.2. Unconfirmed Mode

The unconfirmed mode does not require a downlink ACK from the NS. However, to determine the connectivity loss between the ED and GW, the ED enables ADR ACK bit by sending a MAC command ADRACKReq in the LoRa frame header (FHDR) after 64 (default) UL packets [9]. In such a case, the NS must send an ACK, but not immediately. Furthermore, LoRaWAN utilizes ADR for SF and TP management in confirmed and unconfirmed modes.

## 3. LoRaWAN Meets ML

This section mainly focuses on existing ML methods applied to LoRa and LoRaWAN for efficient resource management (e.g., SF, TP, BW, and CR) for improving LoRaWAN network performance and efficiency. In the remainder of this section, we present the existing ML, DL, and Reinforcement Learning (RL) methods applied to LoRaWAN.

### 3.1. Improving LoRaWAN Performance Using ML

Here, we identify the need for ML and present state-of-the-art methods for enhancing LoRaWAN performance through efficient resource management. These ML methods applied for improving the performance of LoRa and LoRaWAN are shown in Table 4.

#### 3.1.1. Need for Machine Learning

ML is a rapidly growing field with many applications, including wireless communications [115,116,117,118,119]. ML can be utilized to improve the performance, efficiency, and security of wireless networks [120,121]. However, ML is applied in a mathematical model deficit and algorithm deficit cases in IoT scenarios [122]. In LoRaWAN, resource management decision-making is left open to developers and researchers, allowing them to develop intelligent solutions for demanding IoT applications. One approach is to utilize ML for resource management, revolutionizing the optimization of SF, TP, BW, and other important parameters. ML algorithms empower LoRaWAN networks to dynamically allocate resources, predict network traffic, mitigate interference, and optimize energy consumption, thereby enhancing network capacity, reliability, and battery life. With ML-driven insights, operators can proactively plan network expansions, ensure the quality of service (QoS), and achieve self-optimizing networks that autonomously adapt to changing conditions [123,124]. This cutting-edge technology releases the full potential of LoRaWAN, transforming it into an intelligent, adaptive, and efficient IoT infrastructure for a wide range of applications.

#### 3.1.2. Machine Learning: The State-of-the-Art

The existing state-of-the-art ML methods can be classified into supervised and unsupervised.

##### Supervised Approaches

A load-balancing method for dense heterogeneous IoT networks, such as smart city scenarios, was proposed in [125]. The dataset was gathered from a TTN Mapper (mapping the coverage of TTN GWs based on user data) [126] of frequency, DR, latitude, longitude, RSSI, and SNR. These features describe the successful UL packet transmission from ED to the GW. The authors trained different ML techniques, such as Multiple Linear Regression (MLR), Gaussian Naive Bayes (GNB), Linear Discriminant Analysis (LDA), Quadratic Discriminant Analysis (QDA), Decision Tree (DT), Random Forest (RF), Extremely Randomized Trees (ET), and Voting (Ensemble Learning). The classifiers were applied to an urban IoT network, where the simulation results showed an improved packet success ratio (PSR) and reduced energy consumption of a LoRaWAN network.

In [127], an SF allocation scheme using a support vector machine (SVM) and DT to resolve the collision issue in the LoRaWAN network has been proposed. The training dataset was generated using Simulator for LoRa SimLoRaSF [128] (SF), a custom simulator designed for LoRaWAN using Python. The dataset contains the X and Y coordinates of the ED along with successful SF. The input is labeled as successful (if the packet was successfully received at the GW), interfered (when a packet was unsuccessful due to Co-SF interference), and under sensitivity (when a packet is arriving under the required sensitivity threshold at a specific SF). The SVM and DT classifiers are trained for optimal SF allocation. Their simulation results showed that the SVM and DT could efficiently classify the SF, improving the PSR and transmission energy consumption compared to the random SF allocation method. However, the SimLoRaSF does not consider the downlink communication, which is an essential part of the LoRaWAN.

The authors in [129] solve the resource classification problem (e.g., TP) for static EDs in LoRaWAN through various ML techniques, such as RF, SVM, logistic regression (LR), K-nearest neighbor (KNN), LDA, and GNB. The authors used LoRaSim [30,130,131] network simulator for dataset collection, which is designed for LoRaWAN IoT networks based on Python. The ML algorithms were trained on data from previous packet transmissions, such as SF, CR, Nakagami path loss, and the distance between the ED and GW. As a result, every combination of SF and CR pairs has one optimal TP associated with it. The dataset was split into training and testing by 70% and 30%, respectively. From classification results, it was observed that the RF method achieved the highest accuracy of 92.96% compared to other ML techniques. Their simulation results revealed that suitable TP classification leads to a higher PSR than other non-ML methods, such as ADR.

The authors in [132] proposed a combined path loss and shadowing (CPLS) technique, where ML methods such as LR, SVM, RF, and Artificial Neural Network (ANN) were trained on RSSI, ToA, SF, and SNR. The dataset was collected through a testbed utilizing four static EDs in a line-of-sight (LoS) scenario. After removing the outliers and wrong data collected from the sensors, they divided the dataset into training (80%) and testing (20%). To this end, the authors suggested an enhanced ADR for SF and TP allocation to static EDs. The ML methods were evaluated with root-mean-square error (RMSE), achieving up to 1.566 dB and $R^{2}$ up to 0.94. The enhanced ADR results revealed reduced energy consumption by 43% compared to the ADR of LoRaWAN.

The paper [133] proposed an ML approach based on a learning-automata mechanism to extend the lifetime of IoT EDs utilized for forest monitoring. Their proposed approach selects the most energy-efficient ED to act as a cluster head. The simulation results showed that the proposed learning-automata mechanism increased the lifetime of IoT EDs by up to 6.7 times. Their proposed approach has empirically proven that the learning automaton consistently converges on the most suitable ED to serve as the cluster head based on energy consumption metrics, proving their proposed approach is scalable and can easily be adapted to different IoT applications.

##### Unsupervised Approaches

One of the challenges of LoRaWAN is the collision occurring between the packets of EDs when transmitted at the same time with the same SF over the same channel. A K-means clustering algorithm was proposed for reducing collision probabilities [134]. Their proposed K-means are responsible for grouping EDs with similar traffic patterns. EDs belonging to different groups have different priorities, and the channel access for the UL packet transmission is determined by the given priority. Their method showed improved performance in terms of collision probability for class *A* and *B* devices. Their proposed method is simple, scalable, and efficient for reducing collision probabilities in LoRaWAN. Similarly, ED profiling using K-means was utilized in [135] to predict the behavior of LoRaWAN traffic. The authors grouped the EDs using the same SF and packet size and trained DT and Long Short-Term Memory (LSTM) using unsupervised traffic pattern classification methods. Their simulation results showed improved performance in terms of PSR by reducing the impact of interference. However, in a dynamic LoRaWAN network, ED profiling can be time-consuming because resources (e.g., SF and TP) are application-dependent. Therefore, their proposed method is applicable to static LoRaWAN network environments.

A learning-automaton-based ML approach was proposed on the NS in [136] to select between two MAC protocols: TDMA and Slotted ALOHA. The selection of each MAC protocol depends on the network traffic load. If the network traffic load is high, TDMA is chosen for packet transmission to avoid collisions. If the network traffic load is low, Slotted ALOHA is utilized for communication to reduce packet delay. The learning automaton adapts the MAC protocol selection based on the feedback received from the environment. The proposed learning automaton-based ML approach has been evaluated in simulation, showing improved performance in the presence of event traffic.

**Table 4:** ML methods applied for improving the performance of LoRa and LoRaWAN.

Ref.	Year	ML Model(s)	ML Approach	Features	Deployment Platform	Dataset Tool	Application(s)
[125]	2018	MLR, GNB, LDA, QDA, DT, RF, ET, Voting	Supervised	RSSI, SNR	Python tool	TTN Mapper [126]	Smart city
[127]	2019	SVM, DT	Supervised	X and Y coordinates, SF	SimLoRaSF simulator [128]	SimLoRaSF simulator [128]	✗
[129]	2023	RF, SVM, LR, KNN, LDA, GNB	Supervised	SF, CR, path loss, and distance	LoRaSim	LoRaSim	Smart parking
[132]	2023	LR, SVM, RF, ANN	Supervised	RSSI, ToA, SF, SNR	✗	Testbed	✗
[133]	2023	Learning-automata	Supervised	RSSI, ToA, SF, SNR	✗	Testbed	Forest monitoring
[134]	2019	K-means	Unsupervised	Traffic patterns and priority	✗	Simulation	✗
[135]	2020	DT, LSTM	Unsupervised	SF and packet size	✗	Tested	Water metering
[136]	2022	Learning-automata	Unsupervised	Traffic pattern	✗	Simulation	Environmental monitoring
[136]	2022	AR, TFT	Unsupervised	Time-stamp, SNR, duty cycle, number of transitions, SF, frequency, ADR, successful transmission, and failed transmission	✗	ns-3 simulator	Smart home/city

✗ = not mentioned in the referenced paper.

The authors in [137] proposed two AI methods, an autoregressor (AR) model and a temporal fusion transformer (TFT) model, for classifying and detecting LoRaWAN traffic to optimize the LoRaWAN network performance. The authors collected a dataset using the ns-3 simulator, where the EDs are placed in an 8 km circle with a centered GW. They considered all possible criteria as features for EDs, including the inter packets time, SNR, DC, number of UL transmissions, SF, frequency, ADR, successful transmission at the GW, and failed transmission. The simulation was executed for 365 days, resulting in a 100 GB database. The TFT method was utilized to forecast the behavior of the LoRaWAN network, and the AR method to detect the surge in traffic with overall classification precision between 94.50 and 99%. However, the suggested AI methods have not been utilized in the ns-3 for online testing.

### 3.2. Improving LoRaWAN Performance Using DL

We begin by highlighting the importance of DL in the LoRaWAN network. We then delve into the cutting-edge DL methods currently employed for solving the resource management issue to enhance the performance of LoRaWAN networks. These DL methods applied for improving the performance of LoRa and LoRaWAN are illustrated in Table 5.

#### 3.2.1. Need for Deep Learning

DL can improve the performance of LoRaWAN networks by optimizing resource parameters, predicting network traffic, mitigating inter- and intra-interferences, and optimizing energy consumption [138,139,140,141,142,143,144,145,146,147,148]. Furthermore, to dynamically allocate resources to EDs, such as BW, SF, and TP, a DL method can be trained on a large dataset generated using simulation tools (e.g., ns-3 or Matlab) or testbeds. Finally, the trained DL method can be deployed on EDs or network servers (ns-3 or testbed deployments) for efficient resource allocation, improving the performance of the LoRaWAN network.

#### 3.2.2. Deep Learning: The State-of-the-Art

An Extended Kalman Filter (EKF)-based LSTM method based on regression method for predicting collision in LoRaWAN network was proposed in [141]. They generated the dataset for a number of collisions for each 20 min interval using the LoRaSim simulator [131]. For training the LSTM, the dataset was split into 70% and 30% into training and testing and scaled to [0, 1]. They utilized the pre-trained LSTM along with EKF for collision analysis. Their results showed an improved RMSE of 0.9863 compared to other approaches, such as Gated Recurrent Unit (GRU) and Recurrent Neural Network (RNN). Hence, with low RMSE, the LSTM-EKF has reduced the number of collisions in online simulation and yielded better performance. The collision in the LoRa network is directly linked with the SF; hence, SF has not been considered for adaptive configuration. As a result, their proposed LSTM-EKF can lead to underperformance when utilized in a dynamic LoRaWAN network.

The study in [146] proposed DeepLoRa, an environment-aware path loss model, utilizing satellite photos to categorize a land cover using Bi-LSTM accurately. In DeepLoRa, first, each pixel of the picture map was class-labeled, which divided the land cover into two classes, non-line of sight (NLoS, buildings, trees) and LoS (no attenuation), to reflect the actual land-cover type. Second, they divided LoRa lines from an ED to GW into identically sized micro-links. Each was then embedded into a different sequence element based on a land cover map. To determine the Estimated Signal Power (ESP) received by GW, the model integrated the sequences with specific input parameters and anticipated related path loss. For all land cover categories, the accuracy of land cover classification was 97.4% (which can be regarded as a true environment reflection). Furthermore, DeepLoRa performed at least 50% better than other models.

A neural-enhanced LoRa demodulation method (NELoRa) was proposed in [143]. A Deep Neural Network (DNN) was trained on a spectrogram of amplitude and phase. The authors conducted indoor and outdoor experiments, and three metrics, i.e., Symbol Error Rate (SER), SNR Gains, and Battery Life Gain (BLG), were used for the performance evaluation of NELoRa. The evaluations showed that NELoRa could obtain much lower SERs. As a result, the proposed NELoRa brought significant gains in the SNR thresholds compared to the dechirp process, and the highest SNR gain observed was 5.94 dB under $SF_{7}$ and 500 kHz of BW. NELoRa resulted in consistent and higher BLG of 27% compared to the baseline.

The paper proposed a DL method [149] for managing the transmission interval of IoT devices in LoRa networks by utilizing Intel Berkeley Research Lab Data [150]. Using an autoencoder, the proposed method first clusters the IoT EDs concerning their data patterns. Second, a local LSTM prediction model is trained using the Intel lab IoT dataset for each cluster to predict the next transmission interval of each ED involved in communication. The Monte Carlo simulation with the Intel lab IoT dataset showed 31% improved scalability.

The paper [147] proposed a DL method for joint collision detection and resource management (e.g., SF). The proposed work utilizes two DL methods: fully connected neural networks (FCNNs) for collision detection and CNN for SF management. The dataset used to train the DL methods was generated using the SimLoRaSF simulator [127], containing the X and Y coordinates of the ED along with SF. The results showed improved prediction accuracy and energy consumption compared to traditional ML methods such as SVM, DT, and RF.

Recently, the authors in [148] proposed an AI framework for SF classification using GRU. To train the GRU model, the dataset was generated in ns-3 and labeled in two ways:

1. *Group-based SF labeling:* the 6 UL packets, represented with $UL_{1}$, $UL_{2}$, *…*, $UL_{6}$ transmitted with $SF_{7}$ to $SF_{12}$ were organized into one group (*g*). From each *g*, the lowest SF was chosen among successful ACK receptions;
2. *Input sequence labeled with SF:* once the SF labeling of each *g* was completed, the authors generated an input sequence of 20 groups with a corresponding labeled SF.

Their proposed AI framework is comprised of two modes: offline and online. The GRU model is trained based on one-time generated data in the offline mode. After training, a pre-trained (i.e., inference model) is utilized in the online mode, which yields the best SF during real-time simulation. The inference model was utilized on the ED side, where the new data with 20 UL sequences for determining the best SF was generated. Once the UL sequence reached a size of 20, the input was fed into the inference model to predict a suitable SF for the next UL packet transmission. Simulation results showed improved PSR compared to the typical ADR approach. However, the GRU model with two layers comprised 990.71k parameters (i.e., space complexity) and 249.67 Mega Floating-point Operations Per Second (MMac FLOPs, representing time complexity); the proposed model deployed on LoRa devices would be computationally costly. To address this issue, the authors in [114] proposed a DNN with reduced computational 13.54 MMac FLOPs and space complexity 52.9k parameters with the use of six sequences of UL packets during the online mode to lower the convergence period and energy consumption.

### 3.3. Improving LoRaWAN Performance Using RL

Here, we discuss the advanced RL methods utilized to address the resource management challenge and further enhance the overall performance of LoRaWAN networks. The cutting-edge RL techniques are designed to intelligently manage network resources like BW, SF, and TP to maximize efficiency and deliver optimal results. These RL methods improving the performance of LoRaWAN are highlighted in Table 6.

**Table 5:** DL methods applied for improving the performance of LoRa and LoRaWAN.

Ref.	Year	DL Model(s)	DL Approach	Features	Deployment Platform	Dataset Tool	Application(s)
[141]	2020	LSTM, LSTM-EKF	Regression	Collisions	LoRaSim	LoRaSim	Smart city
[146]	2021	Bi-LSTM	Supervised	Pathloss	Testbed	Testbed	Localization
[143]	2021	DNN	Supervised	RSSI and SNR	Testbed	Testbed	Smart parking
[149]	2021	Autoencoder, LSTM	Supervised	Sensory data (temperature, humidity, light, voltage)	Monte Carlo simulation	Intel Lab Data [150]	✗
[147]	2022	SVM, DT, FCNN, CNN, RF	Supervised	X and Y coordinates of the ED, SF	SimLoRaSF simulator [127,128]	SimLoRaSF simulator [127,128]	✗
[148]	2022	GRU	Supervised	ED position, SNR, received power	LoRaWAN ns-3 [151]	LoRaWAN ns-3 [151]	Metering
[114]	2023	DNN, LSTM, GRU, SVM	Supervised	ED position, SNR, received power	LoRaWAN ns-3 [151]	LoRaWAN ns-3 [151]	Pet-tracking and metering

✗ = not mentioned in the referenced paper.

#### 3.3.1. Need for Reinforcement Learning

RL approach has several advantages over traditional methods of optimizing LoRaWAN networks [129,152,153,154,155].

1. RL is a data-driven approach, learning rules and policies from experience by interacting with the network and observing the results;
2. RL is a dynamic approach, adapting itself to environmental changes. LoRaWAN networks are constantly changing owing to ED mobility and the underlying propagation environment. As a result, RL can be used to learn how to optimize the network parameters for these changes, ensuring that the network remains reliable and efficient;
3. RL is a scalable approach; thereby, it can be used to optimize large and complex networks.

RL has been utilized in different IoT applications, such as robotics, game-playing, network policy control, and resource optimization [156,157,158,159]. For example, in LoRaWAN, RL could be used to optimize the resources (e.g., SF and TP) by training agents (i.e., EDs) for making decisions to maximize the overall network efficiency and minimize interference and energy consumption [160,161].

#### 3.3.2. Reinforcement Learning: The State-of-the-Art

Paper [162] proposed an RL approach for optimizing and updating LoRa communication parameters. The authors mathematically modeled the average per-node throughput of LoRaWAN networks by considering the heterogeneity of IoT deployments. The authors utilized the RL method to derive optimal disseminating policies by aiming to maximize the accumulated average per-node throughput. The authors compared their approach to the LoRaWAN ADR mechanism. The authors showed that their approach achieved a remarkable increase in the accumulated average per-node throughput of 147%.

Paper [163] proposed a novel method for resource allocation in LoRaWAN networks. The method used Q-learning, an RL technique, to learn the optimal resource allocation policy for each ED in the network. In the proposed method, the GW acts as an agent of Q-learning, where the Q-reward is based on the weighted sum of the number of successfully received packets in the proposed method. The Q-learning method was evaluated using simulation, and it has improved the average PSR by about 20% compared to a random resource allocation scheme.

**Table 6:** RL methods for improving the performance of LoRa and LoRaWAN.

Ref.	Year	RL Model(s)	Reward Feature(s)	Platform	Application(s)
[162]	2019	Evolution strategies (ES) algorithm	Channel conditions	Simpy [164]	✗
[163]	2019	UCB	Q-learning	✗	Gas and water meters
[165]	2019	UCB	ACK	Matlab	Smart metering
[166]	2020	DQN	PDR, ToA, and power usage	✗	✗
[167]	2020	DQN	Network reliability and the power efficiency	Simulation	✗
[168]	2020	DQN	Mobility, channel conditions, traffic load	Simulation	✗
[159]	2021	Q-learning	✗	✗	✗
[169]	2021	RL	SF Sensitivity	LoRa-MAB [21,170]	Monitoring
[154]	2021	RL-ADR	ToA and energy consumption	ns-3	✗
[152]	2021	RL	DER and SNR	LoRaEnergySim [171]	Industrial monitoring
[153]	2021	DRL	Energy cost	Monte Carlo simulations	Real-time application
[172]	2022	MAB	ACK	LoRa-MAB [21,170]	Metering
[155]	2022	DRL	Number of ED, reliability, energy efficiency	Testbed	✗
[173]	2022	DRL	Collision rate and the packet loss rate of EDs	LoRaSim simulator [131]	✗
[174]	2023	Q-Learning, Boltzmann exploration algorithm	Success probability	MULANE [175]	✗
[176]	2023	Multi-arm bandit	Energy	ns-3 module [151]	✗
[177]	2023	DRL	Collision or packet lost	LoRaSim simulator [131]	✗
[178]	2023	Multi-agent regression model	Number of EDs, distribution of EDs, the traffic pattern of EDs	Simulation	✗

✗ = not mentioned in the referenced paper.

To improve the PSR, the authors in [165] proposed a retransmission method based on an Upper Confidence Bound (UCB) algorithm that is used to solve the multi-armed bandit problem. In [165], first, the ED retransmits a packet with random channel selection and learns the quality of each channel based on a positive ACK reception. Then, after learning the best channel for retransmission, the EDs can retransmit on the highest-rewarded channel. Hence, improving the PSR. Their results showed improved PSR compared to random channel selection schemes.

A deep RL (DRL) method was proposed for the dynamic adjustment of SF and TP to mitigate the collision problem in LoRaWAN [166]. The authors considered the PSR, ToA, and TP of an ED as a reward function. To mitigate the collision behavior of the LoRaWAN network, a deep Q-network (DQN) was deployed at the GW for SF and TP management. As a result, their proposed method improved the PSR by 500% under 100 EDs deployed in a 4.5 km region.

The authors in [167] proposed a multi-agent Q-learning algorithm to dynamically allocate TP and SF to EDs during UL packet transmission in a LoRa network. In a LoRa multi-agent system, each agent represents one LoRa ED, where the ED works together with the environment to determine the best TP and SF allocation policies for every UL transmission. The simulation results demonstrated that the suggested algorithm could greatly enhance the energy efficiency and reliability of LoRa networks.

The authors in [168] proposed a DRL method called LoRaDRL-based on DQN for intelligent resource allocation in dense LoRa networks. The primary aim of the proposed LoRaDRL approach is to learn the optimal policy for allocating channels to LoRa EDs. LoRaDRL assigns resources to the EDs by considering the mobility of EDs, the channel conditions, and the traffic load in the network. LoRaDRL results showed improved PDR under dense deployments and mobile EDs compared to the state-of-the-art resource allocation algorithms, such as LoRaSim [131] and LoRa-MAB [21,170].

An RL algorithm was introduced in [169] for SF and TP optimization to improve energy consumption and PSR. The SF and TP are optimized based on the required level of SF sensitivity threshold of the GW. They implemented the RL algorithm in the LoRa−MAB simulator [21,170], which is based on Exponential Weights for Exploration and Exploitation (EXP3). The results showed an improved PSR and energy consumption compared to the LoRa-MAB algorithm [21]. Similarly, an RL-ADR was proposed in [154] to optimize the SF allocation. The agent is trained using the LoRaWAN ns-3 module, considering the variations in the SNR behavior. In addition, the reward function is based on the ToA and energy consumption. Both ToA and energy consumption are measured from each last received packet at the NS. The RL agent learns the efficient SF allocation policies based on the ToA and energy consumption. The proposed RL-ADR is evaluated in comparison with the traditional ADR mechanism of LoRaWAN, where it showed improved energy consumption owing to the fast response of the trained RL agent for efficient SF allocation during communication by the NS. Furthermore, [152] also proposed an RL-based optimized ADR for SF and TP allocation to EDs. The authors of [152] utilized LoRaEnergySim simulator [171], where the states are data extraction rate (DER) and SNR pairs. Moreover, [152] considers SF and TP pair as actions. The results of [152] showed improved DER compared to the traditional ADR mechanism. Another DRL approach has been proposed for optimal channel and SF assignment to EDs in LoRa networks [153]. Their proposed DRL approach utilizes a DQN to learn the optimal policy for channel and SF allocation. They trained the DQN on a historical data dataset to minimize the grid power consumption while satisfying the QoS requirements of the EDs. The proposed approach is evaluated in Monte Carlo and RL simulations and showed improved performance in assigning a suitable channel to EDs, thereby lowering the energy cost.

The authors of [172] proposed the MIX-MAB algorithm for suitable transmission parameters (i.e., SF) allocation to EDs. In MIX-MAB, LoRa EDs interact with the environment, including GWs, to learn the best actions based on the successful reception of ACK messages. In MIX-MAB, an ED always initiates a UL packet transmission towards GW using an SF. In return, the ED receives an ACK upon the successful reception of the packet on the NS. When the ED receives an ACK, it assigns a reward to that successful SF. As a result, this SF is used for the next UL packet transmission. The MIX-MAB was evaluated in LoRa–MAB simulator [21,170] with one GW located at the center of a disc-shaped cell with a radius of 4.5 km, where 100 LoRa EDs were uniformly distributed, each ED transmits 15 packets/hour. The simulation results showed improved convergence time and PSR compared to the LoRa-MAB algorithm.

The authors of [155] proposed a multi-agent DRL ADR mechanism at the NS side for efficient SF and TP allocation to EDs. The proposed multi-agent DRL ADR mechanism consists of three independent DRL algorithms, one for each slice, replacing the traditional LoRaWAN ADR mechanism for assigning TP and SF to EDs. The SF and TP are allocated to EDs based on the rewards such as the number of ED, reliability, and energy efficiency. Their proposed multi-agent DRL ADR mechanism showed improved energy consumption compared to the traditional ADR. Another approach based on DRL for optimizing the SF allocation is studied to improve the GW capacity of the LoRa network [173]. The proposed approach utilizes a DQN to learn the optimal SF assignment policy for a given network state. The reward is computed based on the collision rate and the packet loss rate of EDs. The authors of [131] performed simulation using the LoRaSim simulator, where the results showed a reduced collision rate by up to 30% compared to the existing Min-airtime and Min-distance based SF allocation approaches [130].

An algorithm called Low-Power LP-MAB (MAB) [179] was designed to configure the transmission parameters (e.g., SF) of ED in a centralized manner to improve energy consumption and PSR. The LP-MAB algorithm works on the NS side by interacting with the ED. The NS transmits ACK upon successful packet reception to the ED, where the ED learns the best SF for the subsequent UL packet transmission based on the received ACK for the previous successful communication on a particular SF. As a result, the simulation results of LP-MAB outperform other approaches in terms of energy consumption and PSR.

A Q-learning approach was proposed in [174], known as the score table-based evaluation and parameters surfing (STEPS) approach. STEPS is responsible for dynamically allocating the required SF for UL transmission based on the success probability of the packet and score table. The simulations were conducted using MULANE [175] simulator for different EDs (e.g., 50, 100, 250, 500, 600, and 750). Initially, during the deployment phase, all EDs utilize the same SF. Their results revealed that their proposed STEPS approach could reduce energy consumption by 24% to 27%. Furthermore, it was realized that this achievement was possible due to a reduction in collisions. In other experiments regarding bi-directional communication, their proposed STEPS approach enhanced the network throughput by 18% in a smaller network, while 33% in a relatively larger network compared to ADR, BADR, and LoRaMAB [170].

The paper [176] proposed a lightweight RL approach for appropriate SF allocation to EDs in a LoRaWAN network. The lightweight RL approach utilizes MAB to learn the trade-off between energy consumption and DR. To ensure feasibility, the authors have integrated explicit MAC commands into their proposed method and implemented them in the ns-3 module [151]. Their extensive simulation results showed that their lightweight RL approach outperforms the traditional ADR in single and multi-GW scenarios regarding PSR and energy consumption, owing to learning the optimal SF for each ED in a given environment.

The authors of [177] proposed an SF redistribution method under limited network resources to improve the ED capacity of the LoRa GW. Their proposed method uses a DRL technique to learn the optimal SF allocation for each node, minimizing the collision rate and energy consumption. Simulation results using LoRaSim [131] showed improved capacity.

Paper [178] proposed a multi-agent regression model to improve network planning in time-slotted communications for LoRaWAN. The proposed agent is based on multi-output regression responsible for predicting the network scalability for a given set of joining EDs. The dataset used for training the agent was generated using a series of simulations, considering the features such as the number of EDs, the distribution of EDs, the traffic pattern of EDs, and the channel conditions. The agent utilizes the dataset to train the multi-output regression model. Once the model is trained, the agent predicts the network scalability for a given joining EDs. The simulation results revealed a 3% reduction in the mean absolute error, indicating that the agent can make accurate predictions.

## 4. Simulators and Frameworks for Dataset Collection

In the existing literature, few works have surveyed the publicly available LoRaWAN network simulators [52,55,73,79,80,180]. Therefore, this section highlights a few LoRaWAN frameworks that have been utilized or can be used for dataset collection to resolve collision and resource management issues in the LoRaWAN network. Furthermore, Table 7 illustrates a comparison of dataset collection frameworks and applicable ML methods based on suggested features for resource classification.

### 4.1. LoRaSim: LoRa Simulator

LoRaSim is based on Python, designed to simulate LoRaWAN collision behavior, and mainly consists of four configurations: (1) it simulates a single GW, (2) supports up to 24 GWs, (3) simulates EDs and GWs with a directional antenna, (4) and comprised of multiple networks [30,130,131].

LoRaSim [131] can be used to study the performance of different LoRaWAN network configurations and to evaluate the impact of interference. The interference model in LoRaSim is comprehensive, considering both co-SF and inter-SF interference. Co-SF interference occurs when two or more packets are sent on the same SF. Inter-SF interference occurs when a packet is sent on a different SF than another packet.

On the one hand, a packet is received correctly if it satisfies three thresholds: the minimum co-SF, the minimum inter-SF, and the minimum SNR. On the other hand, a packet is lost only if the overlap of packets is in the time-critical region of the considered packet. The time-critical region is the part of the packet most important for correct reception.

**Table 7:** Comparison of publicly available LoRaWAN frameworks for dataset collection and applicable ML methods based on the required features for resource classification.

Features	Dataset Collection Frameworks				ML Techniques with Required Features for Learning	
	LoRaSim	SimLoRaSF	LoRaWAN-Sim	ns-3 Module	Applicable ML Techniques	Features for Resource Classification (e.g., SF, TP)
Simulation platform	Python	Python	Python	ns-3	✗	✗
Frequency region	EU-868	EU-868	EU-868	EU-868	✗	✗
Device type	*A*	*A*	*A*	*A*	✗	✗
ADR	✗	✗	✗	✔	RL [21,154,170]	ToA, energy consumption, ACK, PSR [21,154,170]
Propagation loss model	log-distance	log-distance	Okumura Hata	log-distance	RF, DT, SVM, DNN	RSSI, SNR, distance, frequency, LoS, NLoS, Antenna height.
Energy consumption model	✔	✔	✔	✔	SVM, DNN, Ensemble, Naive Bayes, DT	Payload Size, DR, TP, SNR, channel occupancy, distance from GW
Mobility environment	✗	✗	✗	✔	SVM, KNN, LSTM, DNN, RL, Hybrid	RSSI, speed, acceleration, location, trajectory, SNR, time-stamp
Buildings environment	✗	✗	✗	✔	ANN, SVM, GPs, DT	Height, density, material, obstructions, RSSI, NLoS conditions
Interference model	✔	✔	✔	✔	LR, DT, SVM, RF, KNN, RNN	RSSI, CIR, SINR, interference power, type of interference
Channel access method	Aloha	Aloha	Aloha	Aloha	Logistic regression, SVM, DT, KNN, Naive Bayes, DNN, Ensemble	LBT, retransmission limit, DC, channel type, no. of EDs
Confirmed mode (ACK)	✗	✗	✗	✔	Logistic regression, SVM, DT, KNN, DNN, Ensemble	Retransmission count, ACK, PSR, DR, ToA, Link margin, history of packets
Unconfirmed mode (no-ACK)	✔	✔	✔	✔	SVM, DT, KNN, DNN, Ensemble	DR, SNR, RSSI, Link margin, history of packets, PER

✗ = not mentioned in the referenced paper; ✔ = defined in the referenced paper.

LoRaSim is a valuable framework for understanding the behavior and performance of LoRaWAN. It can be used to design and optimize LoRaWAN networks to ensure reliable and efficient communication, as utilized in [29,30,70,141,173,181,182,183,184,185,186,187,188,189,190,191,192,193,194,195,196].

### 4.2. SimLoRaSF: Simulator for LoRa SF

SimLoRaSF is a LoRaWAN simulator that can be used to study the impact of different SFs on network performance [127,128]. It is a Python-based framework that uses a discrete-event simulation model. It works by creating a virtual LoRaWAN network and then simulating the transmission of packets between EDs in the network by keeping a track record of the packet transmission time, the SF, the transmitting source (e.g., ED), the packet size, the duration (e.g., ToA), and status of each transmission (e.g., ACK).

The packet transmission is categorized into three statuses: transmitted, interfered with, or under sensitivity. A packet is considered successful if it is received correctly by the GW. A packet transmission is considered interfered with if it is corrupted by interference from another transmission in the network. A packet transmission is considered under sensitivity if the SNR is too low for the destination node to receive it correctly.

### 4.3. LoRaWANSim: LoRaWAN Simulator

The LoRaWANSim [197,198] is a powerful simulator framework that can be used to study the behavior of LoRaWAN networks under PHY and MAC layer features. The framework provides a flexible simulation environment, allowing users to control PHY and MAC layer parameters. The PHY layer models the PHY transmission and reception of LoRa signals. It implements a complete LoRa transceiver, including the ability to generate modulated signals and perform demodulation tasks. It also considers factors such as interference and multiple GW scenarios. The MAC layer models the data traffic on the LoRaWAN network. It manages channel access, ensuring multiple EDs can share the same channel without interfering. Furthermore, it considers UL and downlink interference occurring over the same channel, DC limitations of 1%, and energy consumption.

### 4.4. ns-3: Network Simulator-3

The LoRaWAN ns-3 module [151,199] is among the most widely used simulators for utilizing ML, offering a wide range of LoRaWAN features, such as bi-directional communication, confirmed and unconfirmed modes, support for the ADR on the ED and NS-sides, buildings, DC limitations on the EU-868 MHz frequency, energy consumption module, etc. Furthermore, other promising features and modules, for instance, ADR in unconfirmed mode [7], mobility patterns [200], blind ADR [113] and ML methods [114,148] have been added by other researchers. However, they are not publicly available. Furthermore, this ns-3 module [151,199] has been extensively utilized in the literature [7,10,27,100,113,114,148,199,200,201,202,203].

### 4.5. Remarks

These dataset collection frameworks offer valuable tools for researchers and practitioners to analyze and optimize LoRaWAN networks. These frameworks enable the study of collision behavior [30], performance evaluation under different SFs, and their impact on the LoRaWAN network performance [127], investigation of PHY and MAC layer features [197], and a complete ns-3 framework for LoRaWAN analysis. The frameworks have been widely used in the literature, demonstrating their effectiveness in understanding and improving LoRaWAN network performance. Among these simulators, the state-of-the-art ns-3 module [151] has been utilized for intelligent SF allocation in the existing literature using DNN and multi-arm bandits approaches in [114,176].

## 5. LoRaWAN Datasets

The LoRa research community lacks a large dataset that can be used to study the resource allocation, interference, collision issues, and behavior of LoRaWAN networks in various conditions. The existing datasets are typically small and specific to a particular network or application owing to a few large-scale deployments of LoRaWAN. In addition, the data collected from LoRaWAN networks are not well-documented, making it challenging to use for research purposes. Despite these limitations, the existing datasets can be utilized to examine a variety of aspects of LoRaWAN networks. For example, the LoRaWAN dataset can be used to study the performance of LoRaWAN networks under different conditions, the impact of resource management on network performance, and the traffic patterns of LoRaWAN EDs. The rest of this section discusses the available datasets designed for different IoT applications.

### 5.1. Localization

A large dataset for LoRaWAN and Sigfox was collected in urban and rural areas from 17 November 2017 to 5 February 2018, which contains the RSSI at each GW, latitude and longitude of the ED, and the SF utilized during data transmission [204]. The authors utilized the dataset in [205] for indoor localization, where the results showed that the mean location estimation error for Sigfox was recorded as 214.58 m and 688.97 m in rural and urban scenarios, respectively. In addition, the mean location estimation error for the urban LoRaWAN dataset was 398.40 m. The datasets presented in [204,205] can be leveraged to evaluate the performance of LoRaWAN-based indoor positioning systems by developing new fingerprinting algorithms. The dataset has been extensively utilized for LoRaWAN-based indoor positioning systems in [206,207,208,209,210,211,212,213]. Similarly, 100 RSSI values were collected for a target node and for the 11 anchors at LoS and NLoS in indoor and outdoor environments for improving the localization through LoRa measurements [214,215].

The authors in [216,217] generated a dataset called the “LoRaWAN at the Edge Dataset (LoED)”. The LoED dataset [218] was collected under an urban scenario for four months, where nine GWs were utilized in central London. Among nine GWs, five were outdoor (line-of-sight (LoS)), four GWS located indoors with limited LoS, and one had no-LoS on the ground. The data was captured for a 2–4 month period generated by smart city applications. Overall, 11,263,001 packets were collected from 8503 unique EDs, comprised of the features such as cyclic redundancy check (CRC), RSSI, SNR, SF, frequency, bandwidth, CR, packet type, device address, etc.

The LoRa RF fingerprinting dataset was collected considering indoor and outdoor scenarios comprising FFT and I/Q samples for indoor localization [219,220,221]. On the one hand, the dataset was collected for five consecutive days for indoor scenarios. During indoor dataset collection (e.g., room, outdoor, and office environments), 25 EDs were utilized, where every ED transmits 10 UL packets with an interval of 20 s. On the other hand, the outdoor dataset comprised four different scenarios regarding the distance: 5 m, 10 m, 15 m, and 20 m away from the GW.

The LoRaWAN performance mainly depends on several factors, including the distance between the ED and the GW, the underlying propagation environment, and the network traffic. To study the LoRaWAN network, the authors in [222] generated a dataset (publicly available, [223]) using 311 outdoor tests for 39 GW, which contains data regarding the long-term behavior of the LoRaWAN channel in Brno, Czech Republic. The dataset was collected under different environments, including urban, suburban, and rural areas, for two months, and included information about the SNR and RSSI concerning the closest GW distance.

The authors of [224] collected a fingerprinting dataset by conducting two studies on indoor and outdoor environments. The first study was conducted at the Brno University of Technology in Brno, Czech Republic, and the second was conducted at the University Politechnica of Bucharest in Bucharest, Romania. The dataset [225] comprises the RSSI information for various GWs using $SF_{7}$, $SF_{9}$, $SF_{10}$, and $SF_{12}$. The study showed reduced positioning accuracy in indoor and outdoor experiments. However, the SF dataset is limited since the authors have used only $SF_{7}$, $SF_{9}$, $SF_{10}$, and $SF_{12}$.

### 5.2. Weather Forecast

Owing to the long range of LoRaWAN, it can often be utilized in outdoor environments. However, the performance of LoRaWAN is greatly affected by weather conditions, such as humidity, temperature, and atmospheric pressure. Therefore, the authors in [226] generated a dataset about the correlation between RSSI and SNR conditions from 8 LoRaWAN EDs and a weather station. The dataset [215] was collected for more than 80 days in a vineyard in Italy, including more than 190,000 records of RSSI, GPS coordinates, temperature, humidity, and pressure data. Researchers can use the dataset to develop new algorithms for studying and improving the performance of LoRaWAN under different weather conditions.

### 5.3. Security Attacks

LoRaWAN networks can be vulnerable to security attacks. Therefore, the study in [227] investigated the use of LoRa metadata to detect the presence of security flaws within the network. The authors in [227] collected a dataset of LoRaWAN transmissions for two months with SNR and RSSI measurements. The data collected included normal, collided, and jammed metadata, where normal values are annotated as $CRC_{OK}$ and DoS attacks were denoted as $CRC_{BAD}$. They utilized various ML methods (e.g., logistic regression, DT, RF, and XGBoost) to predict the presence of jamming and security attacks. Among the ML methods, XGBoost was the most accurate ML method for predicting security attacks, since it was found that SNR and RSSI can be used to pinpoint normal versus anomalous signals.

### 5.4. Signal Quality/Path Loss

When designing a LoRaWAN network, it is essential to consider the path loss between the EDs and the GWs. Several factors, including distance, frequency, and weather, can affect path loss. The authors in [228] presented a LoRaWAN measurement dataset collected in Medellin, Colombia. The dataset [229] was collected for about four months by considering only four EDs, which include information about path loss, the distance between ED and GW, frequency, temperature, relative humidity, barometric pressure, particulate matter, and energy consumption. Furthermore, the authors claimed that leveraging the dataset would enable the estimation of weather-induced variations in path loss for LoRaWAN deployments, leading to enhanced precision in tracking and positioning data and the development of more efficient energy reduction strategies.

The dataset in [230,231] was generated using two GW and six mobile EDs in a 6 × 6 ${\mathrm{km}}^{2}$ urban area in Tsinghua University, Beijing, China. The dataset [232] was collected over four months comprising RSSI, PDR of the transmitted packets, locations of the ED and GW, and timestamps of each measurement.

The dataset [233,234] was collected in indoor and outdoor environments where, during the indoor data collection, the distance between the EDs and the GW varied from 5 to 50 m. The floor map illustrated the walls, doors, and windows between the EDs and the GW. In the outdoor environment, railway stations were used without considering obstacles between the EDs and the GW. The dataset includes features such as the timestamp, the SNR, and the RSSI of the received packet at the GW.

### 5.5. Smart City

The dataset [235,236] is divided into two parts: LoRa parameters and sensor readings. The LoRa parameters dataset contains the timestamp of packet transmission, the channel used in packet transmission, the device extended unique identifier (DevEUI), the SNR, the RSSI, and the frame counter (FCNT). In comparison, the sensor dataset includes data about the measured quantities, such as CO2, sound average, sound peak, motion, light, temperature, humidity, and battery levels. The Smart Campus dataset can be used for various applications, including time-series forecasting and number of people prediction.

### 5.6. Resource Allocation

Mainly, the SF allocation is dependent on the underlying propagation environment, mobility, ED position, and sensitivity. Therefore, in our previous work [114,148], we generated a dataset using the state-of-the-art ns-3 module [151], which is publicly available [237]. The dataset was generated for 10 days with a UL period of 10 min, which mainly comprises ED locations (i.e., X and Y coordinates), RSSI, SNR, the distance between ED and GW, and the ACK status of every UL packet transmitted with each SF. The dataset in [237] can be utilized for resource management (e.g., SF) for static and mobile EDs.

### 5.7. Remarks

In addition to existing datasets, few datasets are publicly available with limited documentation. For example, LoRaWAN traffic analysis dataset [238], outdoor experiments conducted for LoRa RSSI [239,240], LoRaWAN dataset using SF [241], and LoRa time series dataset [242]. Furthermore, apart from [237] dataset, these datasets [205,215,218,221,223,225] are not designed for resource management. However, they can be tested for resource allocation since most datasets include RSSI, SNR, and device location information, which are efficient features for resource management. In conclusion, Table 8 provides additional information regarding the datasets discussed in this section.

## 6. ns-3-Based ML Frameworks

The ns-3 frameworks, mainly designed for ML, can be utilized or integrated with the LoRaWAN ns-3 module [151,199] to enhance the performance of LoRaWAN with ML. These frameworks are discussed in the remainder of this section.

### 6.1. ns-3-AI Framework

Currently, researchers are interested in applying ML techniques to wireless communication networks [115,116,117]. It is owing to most ML techniques heavily relying on open-source TensorFlow and PyTorch ML frameworks. These two frameworks are developed independently and are extremely hard to merge. Moreover, connecting these two frameworks with data interaction is more reasonable and convenient. Therefore, the ns3-AI framework was proposed in [243,244]. The ns3-AI framework provides an efficient workflow between ns-3 and Python-based modules, enabling seamless data transfer and interaction between the two modules. As an example, using the ns-3-AI framework: (a) LSTM has been utilized to predict the channel quality, and (b) RL method for controlling the congestion occurring in TCP [244].

### 6.2. ns-3-gym Framework

The ns3-gym is an open-source RL framework, integrating OpenAI Gym and ns-3 [245]. The OpenAI Gym is a popular and open-source RL toolkit providing an interface for interacting with RL environments. The OpenAI Gym offers predefined environments with well-defined state and action spaces, making developing and comparing RL algorithms easier. It supports various RL methods, allowing researchers from academia to focus on developing new learning algorithms. The ns-3 Gym fills the gap between ns-3 and OpenAI Gym by creating an interface. This interface allows researchers to leverage the capabilities of ns-3 within the OpenAI Gym framework. As a result, such integration enables researchers to apply RL techniques to network scenarios and train RL agents to make intelligent decisions in complex networking environments.

For example, the authors in [246] present two use cases of cognitive radio (CR) transmitters to solve the issue of radio channel selection in the IEEE 802.11 WLAN with external interference [247]. In case 1, the transmitter senses the entire BW, while case 2 is related to data transmission, where the transmitter monitors its channel to avoid collisions by selecting a channel free of interference.

### 6.3. Open Neural Network Exchange (ONNX) Framework

ML could be computationally costly in LoRaWAN owing to its low computational power. As a result, energy consumption increases and thereby reducing the network lifetime. Therefore, DL models can be trained on one-time generated data (using ns-3 or testbed), and the pre-trained model (e.g., inference model) can be utilized in LoRaWAN ns-3 modules [199]. The pre-trained model can be configured using an Open Neural Network Exchange (ONNX) [248]. ONNX is an open-source Application Programming Interface (API) based on the C++ programming language for DL and ML techniques. To use ONNX with ns-3, first, an ONNX-supported pre-trained model using TensorFlow and PyTorch can be generated. Then, the pre-trained model can be imported into ns-3 with the help of the ONNX API. The ONNX API will provide raw data during simulation (similar input samples used during ML model training), which can be used for resource management (e.g., SF or TP). One example of ONNX implementation in ns-3 can be found in [249], where it has been utilized to simulate and model the behavior of an Open Radio Access Network [250].

### 6.4. ns-3-FL: Federated Learning Framework

The ns-3-FL [251,252] is a new framework for simulating FL in a realistic network environment. FL is an ML technique that allows multiple devices to train a shared model without sharing their data. This is useful for privacy- and delay-sensitive applications like healthcare and finance.

**Figure 3:**
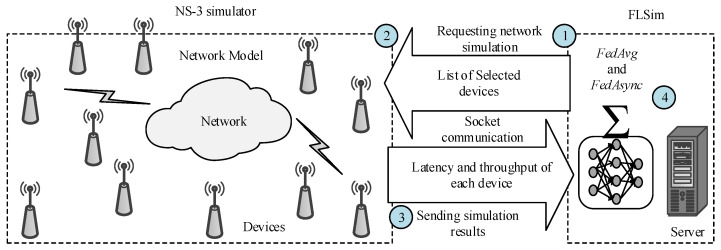
Working procedure of the ns-3-FL framework [252].

The ns-3-FL framework is built on two existing simulators: FLSim [253,254] and ns-3. It provides a realistic and flexible way to simulate FL training and inference in various network settings. FLSim [253] is responsible for data distribution between client–server architecture and FL, whereas ns-3 simulates the network. As an example of ns-3-FL working, (1) the FLSim [253] requests network simulation and selects the number of EDs for the FL training round, (2) the ns-3 performs the simulation for the selected EDs, (3) the ns-3 transmits the latency and throughput of each ED to the FLSim [253], and (4) the FLSim utilizes the received data from ns-3 during computing the convergence time of the global model (using FedAvg and FedAsync algorithms [255,256]) and average throughput for this specified training round, as illustrated in Figure 3.

Furthermore, the ns-3-FL comprises three main components: the learning model, the network model, and the power control.

#### 6.4.1. Learning Model

FL is an ML paradigm that allows multiple clients to train a shared model without sharing their data. The training is achieved by iteratively sending updates to a global model, which is then aggregated by the server using FedAvg and FedAsync algorithms [255,256]. The ns-3-FL supports two types of FL algorithms: synchronous and asynchronous. In synchronous FL, all clients update the model simultaneously, while in asynchronous FL, clients can update the model at different times.

#### 6.4.2. Network Model

The ns-3 network simulator was used to model the latency and throughput between clients and the server, allowing to study of network conditions and their impact on the performance of FL.

#### 6.4.3. Power Model

A power model calculates the energy consumption of FL training. This model considers the number of multiply–accumulate operations performed by the clients and the energy used to transmit the model to the server.

### 6.5. AI-ERA LoRaWAN Framework

**Figure 4:**
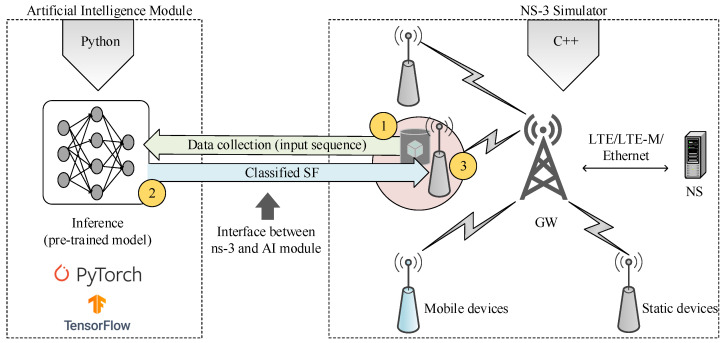
Illustration of the AI-ERA classification framework for spreading factor: (1) inference model deployed on the ED side, where ED prepares input sequence for the model, (2) SF classification by the pre-trained model, and (3) SF adaptation by the ED for uplink packet transmission [114].

Our previously published AI-Efficient Resource Allocation (AI-ERA) framework for SF classification [114,237], designed using AI and ns-3 modules, is a powerful framework, as illustrated in Figure 4. In the AI-ERA framework, the DNN model comprises five fully connected layers, trained using X and Y coordinates, SNR, and received power ($P_{rx}$). After achieving the desired level of classification accuracy, the pre-trained model is deployed on the ED side, where (1) a similar input sequence (utilized during training) is used as input to the pre-trained model, (2) the pre-trained model processes the input and classifies a suitable SF based on the learned knowledge, and (3) a mobile or static ED adapts the classified SF and start transmitting data in UL direction, as shown in Figure 4.

In addition, the AI-ERA framework [237] provides three major components: AI module, dataset, and data labeling.

#### 6.5.1. AI Module

As illustrated in Figure 4, the AI-ERA module is comprised of a pre-trained DNN model, which is deployed on the ED side for efficient SF classification to EDs during simulation.

#### 6.5.2. Dataset

**Figure 5:**
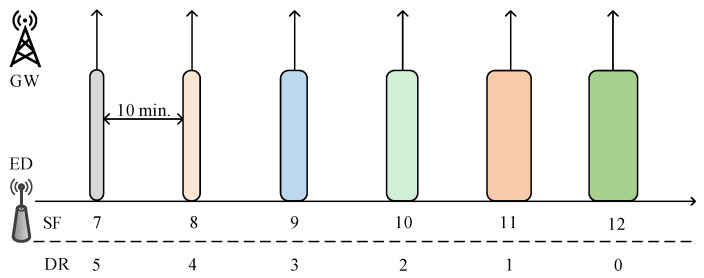
Regular ADR utilized for dataset collection in AI-ERA framework.

During the dataset generation, the AI-ERA framework utilized a regular ADR, where EDs transmit a packet with $SF_{7}$∼$SF_{12}$ at a regular interval of 10 min, as shown in Figure 5. Over a period of 10 days, a dataset was generated with a UL interval of 10 min. The dataset mainly consists of the X and Y coordinates of the ED locations, along with RSSI, SNR, the distance between ED and GW, and the ACK status of each SF in every UL packet transmitted.

#### 6.5.3. Data Labeling

To train the DNN model, the SF was labeled based on the successful ACK reception by the ED. There could be multiple ACK responses for the same packet transmitted with $SF_{7}$∼$SF_{12}$. As a result, the lowest SF is chosen for labeling, and a sequence of six groups as input is fed to the DNN model.

### 6.6. LoRaWAN Bandit Framework

LoRaWAN bandit provides an RL framework that can be utilized to allocate the optimal SF to EDs by leveraging a MAB approach [176,257]. The LoRaWAN bandit framework learns the trade-off between energy consumption for each SF. The framework is designed to work in two phases: exploration and exploitation. During the exploration phase, the framework utilizes all SF combinations and learns how the energy consumption and DR is affected during simulation. In the exploitation phase, the framework selects the optimal SF based on the learned information.

The framework utilizes delayed feedback, where it does not expect immediate feedback. However, it receives feedback after a delay caused by several factors, such as the time it takes to reach the GW and the time it takes to process the packet. The framework is implemented in the ns-3 simulator using the LoRaWAN module [151].

### 6.7. Remarks

The ns-3-based ML frameworks can be utilized by researchers and practitioners to analyze, optimize, and improve the performance of the LoRaWAN networks by efficiently managing the resource parameters. However, these ML frameworks [244,246,248,252] are designed for a different purpose. In addition, the ML frameworks in [114,148,237] and [176,257] are specifically designed for resource management utilizing the state-of-the-art LoRaWAN module [151]. These ML frameworks [114,148,176,237,257] can be adapted to improve the performance of LoRaWAN through resource management.

## 7. Discussion

In the current literature, ML-, DL-, and RL-based solutions have been extensively studied to tackle the critical challenge of resource allocation in LoRaWAN networks, aiming to enhance the performance of the LoRaWAN network. However, existing approaches predominantly focus on resolving resource allocation at the ED level or on the NS, leading to certain limitations and opportunities for improvement.

### 7.1. ML Methods at the ED Side

In the literature, several ML methods have been utilized at the ED side [114,127,148], bringing several advantages. The EDs can make autonomous decisions locally regarding the SF selection without constant communication with the NS, thereby reducing communication overhead. In addition, latency is particularly advantageous in scenarios with limited network bandwidth, unstable connections, and the availability of limited communication channels. Moreover, utilizing ML methods for SF classification can efficiently improve the convergence period under static and mobile IoT EDs, thus improving the packet success ratio [114].

However, utilizing ML methods for resource management on the ED side can be disadvantageous. For example, the primary concern is the computational load it places on resource-constrained EDs. Low-power IoT devices with limited processing capabilities may struggle to support ML models due to the significant computational power and memory requirements. Therefore, extra computational power can lead to excessive energy consumption. Moreover, training and maintaining the models on each ED can be challenging, particularly when EDs have different hardware and software configurations, making it difficult to ensure model consistency across the LoRa network.

### 7.2. ML Methods at the NS Side

When ML methods are utilized on the NS side, it can bring several advantages. For instance, the NS generally has more computational resources, allowing more complex and accurate models to be trained and deployed for resource management. Leveraging a centralized dataset, potentially containing data from massive EDs, can lead to a more comprehensive and accurate ML model for resource classification. Furthermore, updating and maintaining the ML model can be efficiently performed from a centralized location, ensuring consistency across the network.

However, implementing ML methods on the NS can have a negative impact on the network performance. In terms of mobility, if an ED receives a downlink LinkADRReq MAC command from the NS that contains a new SF, and when the ED transmits a packet with the updated SF, it may not be delivered to the GW, resulting in packet loss. This is because the underlying propagation environment changes significantly when the ED is mobile [10,114]. In addition, communication between the EDs and the NS for resource allocation decisions may introduce latency in a large-scale LoRaWAN deployment. Relying on the NS for every resource allocation decision might lead to single points of failure and reduce network performance in terms of increased convergence period.

### 7.3. Remarks

Resource allocation through ML-, DL-, and RL-based solutions has promising potential to enhance the LoRaWAN network performance. However, addressing the challenges associated with dataset quality and considering the trade-offs of implementing the ML method on the EDs and the NS side is essential for developing effective and efficient resource allocation mechanisms in LoRaWAN networks. The choice between the two approaches must be made carefully, depending on the specific requirements and constraints of the LoRaWAN network. Both options present unique advantages and disadvantages that should be thoroughly evaluated for optimal performance and scalability.

## 8. Future Recommendations

This section presents future recommendations for SF and TP allocation to EDs using ML to improve the overall performance of LoRaWAN.

### 8.1. Spreading Factor Classification

The allocation of SF is dependent on few parameters, such as GW sensitivity ($S_{GW}$) [27] and ED sensitivity ($S_{ED}$) [105] thresholds (illustrated in Table 9), the distance between the GW and ED [7], path loss [129,146], ToA [133], interference/collision [201], ED positions with SNR [147,148], retransmission [10], the ratio of UL and downlink ACK [105], and packet drop ratio [258]. However, to apply ML/DL for SF allocation to EDs, multiple parameters have been adopted in the current literature, for example, the received power (i.e., RSSI), SNR, the distance between ED and GW, and ED position [114]. For mobile EDs, the ML methods should be deployed on the ED side since the propagation environment changes drastically. Therefore, the decision of efficient SF selection by ED will help to deliver a packet successfully, thereby improving the packet success ratio. However, deploying the ML on the ED side will increase the computational cost as low-power devices with limited processing capabilities may struggle to support ML models due to the significant computational power and memory requirements. On the other hand, it is recommended to utilize the ML method on the NS side for static EDs. In such a case, the propagation environment remains unchanged [10].

### 8.2. Transmission Power Classification

In LoRaWAN, an ML model can be trained for efficient TP allocation to EDs in an intelligent manner. The input features for TP classification might include RSSI, link quality indicator (LQI), energy consumption, interference in the channel, and distance between the GW and ED. The TP level in LoRaWAN ranges from 2 dBm to 14 dBm and can be divided into five possible classes (2, 5, 8, 11, 14) dBm [152,155,167]. A packet successfully delivered with any SF and TP level can be labeled with a particular TP. For example, a DNN model can be trained to classify an appropriate TP level for the EDs based on the provided inputs.

### 8.3. Multiclass Multioutput Classification

Few existing works have investigated SF and TP classification problems individually. However, both SF and TP classification can be achieved simultaneously, which can be regarded as a multiclass multioutput classification problem, referred to as multi-resource classification (MRC). In the case of SF classification, there are six possible classes (i.e., $SF_{7}$, $SF_{8}$, $SF_{9}$, $SF_{10}$, $SF_{11}$, and $SF_{12}$). Similarly, the TP, ranging from 2 dBm to 14 dBm, can be classified into five possible classes (2, 5, 8, 11, 14) dBm [152,155,167]. During dataset collection, a packet would be transmitted with a pair of SF and TP. Based on the successful ACK reception by the ED for the lowest SF and TP pair, the features such as RSSI, SNR, the distance between ED and GW, ACK status, and X and Y coordinates will be labeled. Based on the requirements of the application (e.g., mobile or static), the ML/DL method can be implemented either on the ED side (in the case of mobile application) or on the NS side (for static application). It is because the underlying propagation environment changes drastically in the case of mobility, thereby the SF and TP selection decision should be taken by ED rather than NS.

## 9. Conclusions

LoRaWAN is a promising IoT protocol for long-range and ultra-low power consumption applications. However, a few challenges need to be addressed before LoRa and LoRaWAN can be widely deployed, such as LoRa parameters configuration, interference, and optimized ADR. One promising approach to overcoming these challenges and improving the performance of LoRaWAN is ML. ML can be used to develop robust, efficient, and intelligent ADRs responsible for resource parameter configurations (e.g., SF, TP, CR, etc.). Furthermore, the field of ML in LoRa and LoRaWAN is growing fast, with recent research and development focusing on various areas such as SF and TP classification, collision analysis, and interference mitigation.

In conclusion, this survey provides a detailed analysis of the state-of-the-art ML/DL/RL applications utilized for improving the performance of LoRaWAN. Further, the survey discusses the publicly available dataset collection frameworks and publicly available datasets. For the identified required features, the use of potential ML methods has been determined for improving the performance of LoRaWAN. Furthermore, the ns-3-based ML frameworks have been highlighted that can be integrated with the widely adopted LoRaWAN ns-3 module. Finally, a discussion on current ML research efforts is highlighted with features utilized for ML, DL, and RL, along with future recommendations that show how the LoRaWAN performance can be further improved using ML techniques.

## Figures and Tables

**Table 1:** Key features of widely adopted IoT technologies [10,11].

Feature	LoRaWAN	Sigfox	NB-IoT	Weightless	LTE-M
Spectrum	ISM band (region-specific)	868 MHz, 915 MHz	1800 MHz, 2100 MHz	915 MHz, 2400 MHz	1800 MHz, 2100 MHz
Bandwidth	125, 250, and 500 kHz	100 Hz	Narrowband, typically 200 kHz	LTE bandwidth, typically up to several MHz	Typically several MHz
Modulation	Chirped spread spectrum	Ultra Narrowband)	GMSK	various (depends on the variant (GFSK)	OFDM
Payload size	Up to 243 bytes	Up to 128 bytes	Up to 1600 bytes	Up to 255 bytes	Up to 1500 bytes
Data rate	Up to 100 kbps	Up to 100 bps	Up to 200 kbps	Up to 1 Mbps	Up to 1 Mbps
Range [km]	Urban = 5, Rural = 20	Urban = 10, Rural = 40	Urban = 1, Rural = 10	Up to 10 km	Up to 10 km
Adaptive data rate	Yes	No	Yes	Yes	Yes
Energy consumption	Very low	Very low	Low to moderate	Low	Low to moderate
Mobility support	Yes (without ADR)	No	Limited	Limited	Limited
Localization	RSSI and TDoA [12,13]	No	No	Yes	Varies
Private network	Yes	No	Yes	Yes	Yes
Bidirectional communication	Yes	No	Yes	Yes	Yes
Deployment	Public	Closed	Public	Public	Public
Simulators [public]	Yes [14,15,16,17,18,19,20,21,22,23,24,25,26,27,28,29,30]	Yes [31,32,33]	Yes [34,35]	Not publicly available	Yes [35,36,37,38]

**Table 2:** Overview of surveys and tutorials on LoRa and LoRaWAN from 2018 to July 2023.

Ref.	Year	Main Focus of Survey	Brief Description of Main Topics Covered
[43]	2018	Pros and Cons of LoRaWAN	Compared existing solutions along with pros and cons and highlighted challenges and solutions.
[44]	2018	Security risks of LoRaWAN	Discussed and analyzed the impact and likelihood of each security threat in LoRaWAN.
[45]	2018	LoRa and its applications	Presented a comprehensive review regarding LoRa and its applications.
[46]	2019	advantages and disadvantages	Compared NB-IoT, LoRa, and Wi-Fi HaLow in terms of their main characteristics.
[47]	2019	Comparative study	Studied LoRaWAN, NB-IoT, LTE-M, and Sigfox and their use in WSN scenarios.
[48]	2019	Edge and Fog computing	Discussed LoRa-based edge and fog computing paradigms, highlighted pros and cons.
[49]	2019	LoRa/LoRaWAN challenges	Reviewed challenges of LoRa in scalability, capacity, and signal collision.
[50]	2019	Overview of LoRaWAN, DASH7, and NB-IoT	Reviewed the architectures and addressed the mobility management issues, and presented a comparative study of LoRaWAN, DASH7, and NB-IoT.
[51]	2019	Overview LPWA technologies	Provided an overview of the existing solutions and identified key research challenges to be addressed in LPWA technologies.
[52]	2019	LoRaWAN simulators	Reviewed several existing available simulators for LoRa/LoRaWAN along with design requirements and their limitations and how to improve simulators.
[53]	2019	Security and energy	Overviewed LoRaWAN in terms of security and energy based on existing state-of-the-art.
[54]	2019	Capacity of LoRaWAN	Studied the capacity of LoRaWAN in terms of ADR, channels, SF, RF, and ED density, along with challenges and future research opportunities.
[55]	2019	LoRaWAN simulators	Provided an overview of existing simulators for LoRa/LoRaWAN with requirements and limitations.
[56,57]	2020	ADR optimization	Reviewed the existing ADR solutions, discussed the impact on the performance of LoRaWAN networks, identified challenges and future optimization techniques for improving the ADR.
[58]	2020	LoRa networking challenges	Investigated the challenges in terms of networking faced during deployment, presented recent solutions, and discussed open issues considering practical large-scale deployment of LoRa networks.
[59]	2020	Feasibility of adapting UDN	Carried the feasibility of adapting an ultra-dense network (UDN) within LoRaWAN and provided details of Mesh-LoRaWAN topology for UDN.
[60]	2020	Visual data transmission	Evaluated existing techniques regarding the image transmission over LoRa networks, presented challenges, and solutions to overcome them.
[61]	2020	LoRaWAN mesh networks	Presented a review and comparative analysis on the classification of multihop communication solutions, discussed issues and highlighted future research directions.
[62]	2020	Security in LoRaWAN	Analyzed security issues and possible network attacks in LoRaWAN and presented countermeasures preventing LoRaWAN from attacks.
[63]	2020	Routing in LoRaWAN	Discussed related approaches concerning multihop communication and routing protocols.
[64]	2020	Confirmed traffic in LoRaWAN	Highlighted use cases, examined several aspects of confirmed traffic along with existing solutions.
[65]	2020	Performance review of LoRa	Discussed the LoRa technology and reviewed performance.
[66]	2021	Use of ML in LoRa	Surveyed the general issues related to LoRaWAN, overviewed the ML solutions, and highlighted key future research directions.
[67]	2021	LoRaWAN optimizations	Presented existing solutions in five aspects: coexistence, resource allocation, MAC layer, network planning, and mobility support.
[68]	2021	UAV-Based LoRa communication	Studied deployments of UAV-based LoRa network and reviewed systematically focusing on the communication setup and its performance.
[69]	2021	ADR enhancements	Reviewed the existing ADR solutions with regard to mobility.
[70]	2021	Routing in LoRaWAN	Investigated routing approaches in multi-hop networks.
[71]	2021	Comparative analysis LoRaWAN and NB-IoT	Compared LoRaWAN and NB-IoT in terms of power consumption, security, latency, and throughput perspectives.
[72]	2021	Performance evaluation	Studied the factors affecting the capacity of the LoRa networks and its performance.
[73]	2021	Simulation tools	Presented the available simulation tools utilized for LoRaWAN performance assessment in ns-3.
[74]	2022	Resource allocation (e.g., SF)	Presented a concise overview of the traditional SF assignment methods to IoT end devices.
[75]	2022	LoRaWAN optimizations	Discussed various aspects, including bandwidth, modulation, data rate, coverage, link budget, payload, power efficiency, security, ADR optimizations, and localization concerning LoRaWAN.
[76]	2022	LoRa networking techniques	Surveyed the LoRa network techniques in LoRa (PHY layer), LoRaMAC layer (WAN), and application layers along with challenges and future trends.
[77]	2022	LoRaWAN protocols	Provided an extensive survey on the existing LoRaWAN communication protocols focusing on the energy efficiency at both LoRa (PHY layer) and LoRaMAC layer (WAN).
[78]	2022	Energy efficiency	Surveyed the existing works on energy efficiency at LoRa and LoRaWAN.
[79,80]	2022	LoRa simulators	Presented a comparative study simulation tool for the simulation of LoRa/LoRaWAN networks.
[81]	2022	LoRaWAN security	Highlighted vulnerabilities and security attacks, discussed their systematic mitigation approaches.
[82]	2022	Recent advancement in LoRa	Reviewed LoRa concerning analysis, communication, security, and applications.
[83]	2023	Scalability in LoRaWAN	Discussed scalability challenges with existing state-of-the-art solutions to assist LoRaWAN deployment in massive IoT networks.
[84]	2023	Artificial Intelligence of Medical Things (AIoMT)	Explored the current literature in the AIoMT, emphasized the powerful association between AI and IoT technologies.
Our survey		ML for resource management (e.g., SF, TP, BW)	This survey presents an in-depth review of resource management issues with state-of-the-art ML solutions, discussing the LoRaWAN frameworks for dataset collection, providing a constructive review on the ns-3-based ML frameworks, and presents future recommendations.

**Table 3:** Time-on-air, duty cycle, and fair access policy (FAP) conditions at 125 kHz and packet size of 51B [101,102].

Conditions	SF7	SF8	SF9	SF10	SF11	SF12
ToA [ms]	118.0	215.6	390.1	698.4	1478.7	2793.5
1% DC [s]	11.8	21.6	39.0	69.8	147.9	279.3
1% DC [msg/h]	305	167	92	51	24	12
FAP [avg·s]	339.9	620.8	1123.6	2011.3	4258.5	8045.2
FAP [avg/h]	10.6	5.8	3.2	1.8	0.8	0.4
FAP [msg/h]	254	139	76	42	20	10

**Table 8:** Summary of existing datasets utilized for different applications of LoRaWAN.

Year	Paper Ref.	Tool Used	Dataset Variables	Size of Dataset	Purpose	ML Method
2018	[205]	Testbed	SF, RSSI, ED positions [204]	123,5229	Localization	KNN
2019	[215]	Testbed	RSSI [214]	✗	Localization	Least squares
2020	[218]	Testbed	CRC, RSSI, SNR, SF, frequency, bandwidth, coding rate, packet type [216]	11,263,001	Smart city, capacity planning	✗
2021	[221]	Testbed	IQ/FFT [220]	✗	Localization	CNN
2021	[223]	Testbed	SNR, RSSI, Frequency [222]	✗	✗	✗
2022	[225]	Testbed	RSSI [224]	Outdoor = 16,054, indoor = 7752	Localization	KNN, LR, DT, SVM
2022	[215]	Testbed	RSSI, GPS coordinates, along with weather data [226]	190,000	Weather forecast	✗
2022	[227]	Testbed	RSSI, SNR, CRC	✗	Security analysis	Logistic regression, DT, RF, and XGBoost
2022	[229]	Testbed	Timestamp, ED id, energy consumption, SF, SNR, distance, frequency, and other sensory data [228]	930,753	Positioning, energy behavior of LoRa	Linear regression
2022	[232]	Testbed	Timestamp, RSSI, PDR, locations of ED and GW [230,231]	✗	Tracking	✗
2023	[234]	Testbed	Timestamp, SNR, RSSI [233]	✗	Locomotion mode recognition	Zero-Shot learning
2023	[236]	Testbed	Timestamp, SNR, RSSI, frame counter, and other sensory data [235]	✗	Smart city, people counting	KNN, LSTM.
2023	[237]	ns-3 [151]	RSSI, SNR, ED positions [114]	108,401	SF management	DNN, LSTM, GRU

✗ = not mentioned in the referenced paper.

**Table 9:** Sensitivity and required SNR of EDs and GW with 125-kHz mode [27,200].

SF	GW Sensitivity ($S_{g}$) [dBm]	ED Sensitivity ($S_{e}$) [dBm]	SNR [dB]
12	−142.5	−137.0	−20
11	−140.0	−135.0	−17.5
10	−137.5	−133.0	−15
9	−135.0	−130.0	−12.5
8	−132.5	−127.0	−10
7	−130.0	−124.0	−7.5

## Data Availability

Not applicable.
